# Employing Hybrid Lennard-Jones and Axilrod-Teller Potentials to Parametrize Force Fields for the Simulation of Materials’ Properties

**DOI:** 10.3390/ma14216352

**Published:** 2021-10-24

**Authors:** Danilo de Camargo Branco, Gary J. Cheng

**Affiliations:** 1 School of Aeronautics and Astronautics, Purdue University, West Lafayette, IN 47907, USA; dbranco@purdue.edu; 2 School of Industrial Engineering, Purdue University, West Lafayette, IN 47907, USA

**Keywords:** pure metals, simple alloys, MXenes, force field, many-body, parametrization

## Abstract

The development of novel materials has challenges besides their synthesis. Materials such as novel MXenes are difficult to probe experimentally due to their reduced size and low stability under ambient conditions. Quantum mechanics and molecular dynamics simulations have been valuable options for material properties determination. However, computational materials scientists may still have difficulty finding specific force field models for their simulations. Force fields are usually hard to parametrize, and their parameters’ determination is computationally expensive. We show the Lennard-Jones (2-body interactions) combined with the Axilrod-Teller (3-body interactions) parametrization process’ applicability for metals and new classes of materials (MXenes). Because this parametrization process is simple and computationally inexpensive, it allows users to predict materials’ behaviors under close-to-ambient conditions in molecular dynamics, independent of pre-existing potential files. Using the process described in this work, we have made the $Ti_{2}C$ parameters set available for the first time in a peer-reviewed work.

## 1. Introduction

Molecular Dynamics (MD) simulations require the use of force field parameters or files containing a formatted list of energy states and electrical charges that are used for calculations of atomic systems energy, forces, velocities, and positions. Each class of materials has an appropriate type of force field since the force field model type specifically determines the energy of a system where a type of interatomic interaction dominates. MD also allows using hybrid force fields if mixed types of interactions are present within a system.

Modeling noble gases can be done by employing simple 2-body Van der Walls interaction [1]. Lennard-Jones is between the simplest 2-body interaction models which can simulate noble gases. It has a strong repulsive term dominant at short interatomic distances, and a smooth attraction term preponderant at longer interatomic distances. For noble gases, this kind of modeling is sufficient and other sorts of bonding terms are not considered for the system’s potential energy calculation (Equation (1)).

(1) $$E=\underset{i}{\sum }\underset{j>i}{\sum }4\epsilon \left[{\left(\frac{\sigma }{r_{\mathrm{ij}}}\right)}^{12}-{\left(\frac{\sigma }{r_{\mathrm{ij}}}\right)}^{6}\right]{,\;r}_{\mathrm{ij}}{<r}_{c}$$

where ϵ is the parameter related to the pair-interaction energy, σ is the zero-crossing distance for the potential energy pair-wise interaction, $r_{\mathrm{ij}}$ is the interatomic distance between atoms i and j, and $r_{c}$ is the cutoff radius above which the pair-wise energy is truncated.

Metallic bonding is related to clouds of electrons that move between the conduction and valence bands. Hence the embedded-atom method (EAM, EAM FS—Finnis-Sinclair, EAM ALLOY, and ADP—Angular Dependent Potential) and modified embedded-atom method (MEAM) force fields are suitable for metallic system’s energy determination. These force fields discretize the space around a reference atom. At each point in the discrete space, the effective charge, used for the pair potential term’s determination ($\Phi_{\alpha \beta }$), and electron density ($\rho_{\beta }$), used for the embedding energy function’s calculation ($F_{\alpha }$), are listed and used for the system’s potential energy determination (Equation (2)) [2].

(2) $$E=\underset{i}{\sum }\underset{j>i}{\sum }\left(F_{\alpha }\left(\sum \rho_{\beta }\left(r_{\mathrm{ij}}\right)\right)+\frac{1}{2}\sum \Phi_{\alpha \beta }\left(r_{\mathrm{ij}}\right)\right)$$

The EAM force field files must follow a strict format with header lines containing the atomic number, mass, lattice constant, lattice type, number of discretization points for embedding function values and effective charge tabulation, density step, distance step, and interatomic distance cutoff. Below the header, a table of embedding function values, followed by effective charge and electron density function values, is provided. The detailed format can be found in the LAMMPS (Large-Scale Atomic/Molecular Massively Parallel Simulator—Molecular Dynamics simulations software) manual documentation [3].

EAM and MEAM are different because EAM force field files present a list containing predefined values for embedding energy, electron density, and effective charge for a single element, while the MEAM force field parameters are determined through an extensive and complex mathematical formulation. MEAM files can be simplified by neglecting several parameters. However, these assumptions must be done carefully, so the force fields model still appropriately fits the system’s energy states.

The MEAM force fields require two types of files. The library file, which contains energy calculation parameters for each element, as if the system was composed only by that element. The main MEAM file contains parameters for the embedding energy and the pair potential interaction functions. The embedding function formulation details can be found in the Sandia Report for Molecular Dynamics Simulations [4]. This force field parametrization can be complex and computationally expensive due to the number of parameters involved to produce accurate models for potential energy calculations. Modeling employing the MEAM force fields can generate significant errors since it is not hard to make an incorrect assumption during the parametrization process, which may drastically interfere with the final simulation results. Also, a MEAM file may be created to determine a specific material property, being inaccurate for other unrelated properties. We consider that using this force field is a good option when material properties are known beforehand since simulation verifications can be done when a comparison baseline is available. Hence the model’s parameters can be adjusted to approach the experimental values. It may not be a good option for a novel metallic system that is being developed from scratch and for which material properties are still unknown.

Materials that present covalent bonding can be modeled employing the ReaxFF. This force field considers the bond order in the covalent bond and uses it for the system’s energy determination. ReaxFF considers the energy contributions of different types of interactions to compute the system’s total energy (Equation (3) [5]).

(3) $$E_{\mathrm{system}}{=E}_{\mathrm{bond}}{+E}_{\mathrm{over}}{+E}_{\mathrm{under}}{+E}_{\mathrm{val}}{+E}_{\mathrm{pen}}{+E}_{\mathrm{tors}}{+E}_{\mathrm{conj}}{+E}_{\mathrm{vdWaals}}{+E}_{\mathrm{coulomb}}$$

where $E_{\mathrm{bond}}$ is the bond energy term; $E_{\mathrm{over}}$ and $E_{\mathrm{under}}$ are respectively the over and under coordination obtained as the difference between the bond order and valence values for each atom in the system; $E_{\mathrm{val}}$ is the energy contribution from the valence angle terms; $E_{\mathrm{pen}}$ is the stability-related term for systems where atoms contain two double bonds sharing the valence angle; $E_{\mathrm{tors}}$ is the energy term related to the torsion angle; $E_{\mathrm{conj}}$ is the energy contribution due to the conjugation effect; $E_{\mathrm{vdWaals}}$ is the non-bonded van der Waals energy term; and the $E_{\mathrm{coulomb}}$ is the energy term related to Coulombic interactions between atom pairs. More details about these terms were described by Van Duin et al. (2001) [5].

ReaxFF requires extensive and detailed considerations to generate a valid force field able to simulate interatomic interactions into a system where atoms are covalently bonded. Although detailed, ReaxFF can still show considerable deviations from quantum mechanics results [6,7].

Organic molecules and their interactions can be modeled with AIREBO [8] force fields which consider the Reactive Empirical Bond Order ($E_{\mathrm{ij}}^{\mathrm{REBO}}$) energy term, the 2 ($E_{\mathrm{ij}}^{\mathrm{LJ}}$), and 4 ($E_{\mathrm{kijl}}^{\mathrm{TORSION}}$) body interactions energy terms, as shown in Equation (4), where the indices i, j, k, and l represent atoms i, j, k, and l in the atomic system.

(4) $$E=\frac{1}{2}\underset{i}{\sum }\underset{j\neq i}{\sum }\left[E_{\mathrm{ij}}^{\mathrm{REBO}}+E_{\mathrm{ij}}^{\mathrm{LJ}}+\underset{k\neq i,j}{\sum }\underset{l\neq i,j,k}{\sum }E_{\mathrm{kijl}}^{\mathrm{TORSION}}\right]\;$$

Ionic compounds can be modeled using Buckingham force fields with a Coulombic term, which can be semi-empirically parametrized using ab initio calculations and calibration using the experimentally determined melting point of the material [9]. Pedone et al. [10] use a free energy minimization strategy tailored by material properties to parametrize their system’s energy model in Equation (5).

(5) $$E=\underset{i}{\sum }\underset{j\neq i}{\sum }\frac{z_{i}z_{j}e^{2}}{r_{\mathrm{ij}}}{+D}_{\mathrm{ij}}\left[{\left({1-e}^{{-a}_{\mathrm{ij}}\left(r_{\mathrm{ij}}{-r}_{o}\right)}\right)}^{2}-1\right]+\frac{C_{\mathrm{ij}}}{r_{\mathrm{ij}}^{12}}$$

where e, in the first term, is the electron charge; $z_{i}$ is the electric charge of the atom i; $D_{\mathrm{ij}}$ is the bond dissociation energy; $a_{\mathrm{ij}}$ is a function of the slope of the potential energy well; $r_{o}$ is the equilibrium bond distance; and $C_{\mathrm{ij}}$ is the coefficient for the repulsive contribution used for proper modeling at high temperatures and pressures.

Like this work, recent force field parametrization techniques [11,12,13,14] have been developed to simplify the parametrization process’ complexity and reduce its computational cost. The most important aspect of a force field parametrization is its capability to accurately determine the system’s energy variations, atomic forces, and velocities. Independent of the fact that simplifications are made, if the force field can replicate the material’s behaviors, mechanical properties, thermal properties, phase evolutions, etc. it should be deemed appropriate for its reference material. Innumerous works have employed force fields that are not a natural choice for the class of materials they study. Filippova et al. [15] performed parametrizations using the Lennard-Jones interatomic interaction model for determining pair interactions between iron atoms and other metals, instead of using EAM or MEAM. Pratt et al. [7] performed nanoindentation MD simulations using different force field types and showed how force fields that would not be a natural choice for FCC aluminum could be applicable for it.

Force fields that are semi-empirically determined have their parameters’ determination guided by a specific material property or expected experimental phenomena [9]. Therefore, other material properties not directly connected to the one that guided the parametrization may have simulated values significantly deviating from the experimental ones. Materials with reduced size and stability under ambient conditions, such as 2D MXenes, have force fields for which semi-empirical parametrization is challenging [7].

In this work, we focus on a fully computational parametrization of force fields that can determine novel materials’ energy states in which metallic and ionic interatomic interactions predominate. Although titanium carbides present predominantly ionic interactions [16], Osti et al. [17] developed a ReaxFF model for titanium carbides, which shows close agreement with the few experimentally determined material properties for MXenes [18]. Before and even shortly after this force field was developed for the MXenes, Borysiuk et al. [19] and Borysiuk et al. [20] parametrized the force field employing a hybrid pair style consisting of 2 and 3 body interactions for the C-Ti interactions, and EAM for Ti-Ti interactions. This force field resulted in material properties that were approximate to the ones determined via first-principles calculations. However, imperfections remain in this approach since force field hybridizations do not consider the atoms embedding effect in the system, which changes the electron density field.

Using a Lennard-Jones-based 2-body interaction term (Equation (1)), and an Axilrod-Teller 3-body term (Equation (6)), one can simulate the attraction and repulsion terms from the pair of atoms and consider the 3-body angular stability that the Axilrod-Teller term provides. This combination can closely represent an atomic system while no significant phase changes and defects density happen. Defects and phase changes would affect the total energy calculation since the simulations with the LJ + AT parameters set are reliable for the atomic configuration of the phase employed in the parametrization.

(6) $$E_{\mathrm{ijk}}=\frac{Z\left({1+3\cos\theta }_{i}{\cos\theta }_{j}{\cos\theta }_{k}\right)}{{\left(r_{\mathrm{ij}}r_{\mathrm{ik}}r_{\mathrm{jk}}\right)}^{3}\;}$$

where Z is the Axilrod-Teller coefficient to be parametrized; $\theta_{i}$ is the angle formed by an atoms triplet (atoms i, j, and k) between the directions that join the atoms i and j, and atoms i and k; and $r_{\mathrm{ij}}$ is the distance between atoms i and j.

It is important to notice that although all these previously described force fields focus on modeling specific interatomic interactions, none of them can accurately predict all states. Those models are based on assumptions, fittings, truncations, and they are used in MD simulations that do not represent a continuous timeline of events within a system of atoms. Additionally, some force fields parametrizations rely on DFT (Density Functional Theory) calculations that, although more accurate than MD, provide reliable results for a large number of K points in the reciprocal space and appropriate cutoffs.

A simplified force field to represent interatomic interactions for materials as pure metals, alloys, and even compounds having covalent or ionic bonding, as well as metallic bonding, considers a hybrid many-body potential combining both 2 and 3 body interactions. The potential energy function for interatomic interactions in Equation (7) would be truncated after its second term.

(7) $$\Phi \left(r_{1}{,\;r}_{2}{,\ldots ,r}_{N}\right)=\overset{N}{\underset{i<j}{\sum }}E_{\mathrm{ij}}\left(r_{i}{,r}_{j}\right)+\overset{N}{\underset{i<j<k}{\sum }}E_{\mathrm{ijk}}\left(r_{i}{,r}_{j}{,r}_{k}\right)+\ldots +\overset{N}{\underset{i<j<k<\ldots <N}{\sum }}E_{\mathrm{ijk}\ldots N}\left(r_{i}{,r}_{j}{,r}_{k}{,\ldots ,r}_{N}\right)\;$$

where $E_{\mathrm{ijk}\ldots N}$ is the N-body energy term.

One of the simplest force fields that can use truncation combines 2 and 3 body interactions by employing Lennard-Jones and Axilrod-Teller models (LJ + AT). The simplicity comes from the fact that these models only have 2 different parameters for each atomic pair interaction (∈ and  σ in Equation (1)), and one parameter for each atomic triplet set interaction (Z in Equation (6)). The models chosen for our parametrization process aim to make this process as simple and computationally inexpensive as possible, so our parametrization scheme can be an option for many materials researchers. We parametrized the force fields for solid crystalline atomic systems with simple atomic structures containing a low number of different interaction types where bonds between pairs of atoms have a fixed bond order, and interatomic interactions, and system energy can be determined using up to 3-body interactions. Therefore, chain structures, like long organic chains, that require dihedral interactions can’t have their force fields model determined by our parametrization process. This parametrization scheme has the advantages of parameter determination ease, since a relatively low number of ab initio simulations need to be conducted, compared to EAM force field parametrization schemes, reduced number of parameters need to be determined compared to other parametrization schemes, parameters’ determination low computational cost, and fast running time, although EAM running times were reduced compared with LJ + AT running times. Disadvantages of the presented parametrization scheme are that it is valid for specific structures, presenting a low transferability when compared to other parametrization schemes that take into consideration many atomic configurations from the configuration space. Our parametrization scheme is more effective at restricted temperature and pressure states, close to the equilibrium configuration and to the configurations where the self-consistent field (SCF) calculations were performed, and where the electron density field is known. For simulations executed at different conditions, when different phases and defects are present, multiple parametrizations may be needed to improve transferability, making the parametrization more complex and computationally expensive. Another aspect to consider when using the parametrization scheme presented in this work, is the accuracy of simulation results. Since our parametrization scheme only relies on ab initio calculations results, no experimental data is used to weight the parameters fitting and reduce errors with respect to experimental results. It is known that ab initio calculations results may deviate from experimentally determined values. Therefore, we acknowledge this limitation and test how our force field model performs compared to models that take experimental values into account. The force field parametrization scheme proposed in this work should be applied in the absence of other more appropriate potentials, which can accurately predict the material’s states when phase changes happen, and when appropriate experimental data to optimize the model’s parameters fitting is not available. It should be used at the development stage of a material when fast progress is needed to obtain a first approximation of the material’s properties.

Recent developments in materials research, especially with the advent of evolutionary algorithms, that can predict materials crystal structures feasibility, like USPEX (Universal Structure Predictor: Evolutionary Xtallography) [21], are moving the materials field at a fast pace. Therefore, materials characterization even before their manufacturing, when a simplified force field model is available, and little to no experimental data was obtained, is an important factor in this ever-growing field.

The parametrization process’ applicability was tested on pure aluminum, and nichrome, for their structural simplicity and for the fact that these materials have multiple force field files available in the literature. We could use these force fields for mechanical and thermal properties determination through strain and temperature-increasing simulations. The results obtained in these simulations were benchmarked against the simulation results using our Lennard-Jones combined with Axilrod-Teller force field parameters. Additionally, we performed the force field parametrization to the $Ti_{2}C$ because it is the simplest titanium carbide from the MXenes family, which is a novel materials family that is currently being studied by multiple research groups. However, the force fields for this materials family are still not openly available for scientists that want to use them in their MD simulations. Therefore, we considered the $Ti_{2}C$ an appropriate material for the proof of concept of our parametrization process, which can make MD simulations more accessible to researchers who study novel materials like MXenes.

## 2. Materials and Methods

### 2.1. First Principles Calculations

The development of new potential parameters when the experimental determination of material properties is not readily available can take advantage of Quantum Mechanics (QM). In QM, one can obtain a good approximation of an atomic system’s energy state. The first step to determine the energy of an atomic system is choosing the pseudopotential to perform optimizations and SCF calculations. All quantum mechanics simulations in this work employed Ultrasoft pseudopotentials with Perdew-Burke-Ernzerhof (PBE) exchange-and-correlation functional. For aluminum, the charge cutoff was 225.000 Ry and the wavefunction cutoff was 28.532 Ry. For Nickel, the charge cutoff was 475.814 Ry and the wavefunction cutoff was 74.643 Ry. For Chromium, the charge cutoff was 456.606 Ry and the wavefunction cutoff was 48.034 Ry. For Carbon, the charge cutoff was 326.261 Ry and the wavefunction cutoff was 40.187 Ry. For Titanium, the charge cutoff was 575.452 Ry and the wavefunction cutoff was 51.678 Ry. Those cutoff values were automatically filled by the BURAI software (a graphical user interface for the Quantum Expresso [22] software-a quantum mechanics simulation software), for the chosen pseudopotentials, with values that allowed accurate determination of energy states at a low computational cost.

**Figure 1:**
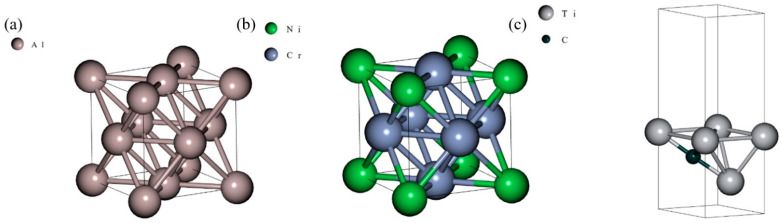
Atomic structure in QM simulations. (**a**) Aluminum, (**b**) Nichrome, and (**c**) ${\mathrm{Ti}}_{2}C$ MXene.

**Figure 2:**
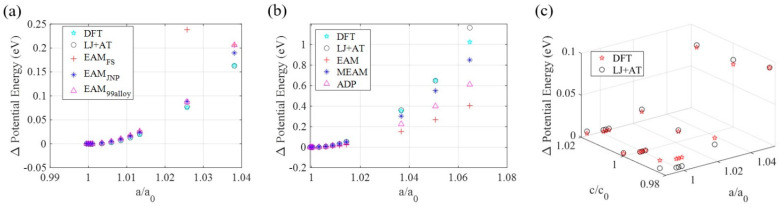
Potential energy variations for QM SCF (DFT) calculations and MD simulations using: (**a**) EAM potential files and the parameters set proposed in this work for aluminum; (b) EAM, MEAM, and ADP potential files, and the parameters set proposed in this work for nichrome; (**c**) the LJ + AT force fields for the ${\mathrm{Ti}}_{2}C$ MXene.

Then, the lattice constants of the simulation cells are set as well as the atomic positions (Figure 1), although the BURAI software displays more atoms due to the symmetry and periodicity of the atomic system. Choosing an appropriate number of K points, which allows good energy determination accuracy and low computing cost (5 in this work for all dimensions), and allowing the simulation cell to relax, one can run atomic positions and a lattice parameters optimizations. These optimizations consist of energy minimization to obtain the atomic system’s ground state. Parameters related to interatomic distances (σ) can then be set in the Lennard-Jones + Axilrod-Teller force field model at a 0 K, 0 bar condition. From this starting point, we altered the lattice parameter, by changing the atomic positions and the simulation cell dimensions. We limited the lattice parameters variations to values (<4% for aluminum, <7% for nichrome, and <4% for $Ti_{2}C$) for which there was enough energy variation that would allow us to determine the force field parameters without significant error effects from a model fitting in a data set with low variation. We then proceeded with the SCF calculations through which we obtained the energy of the different atomic states, at different lattices (Figure 2), so we had energy values to discover the energy-related parameters ϵ and Z for the LJ + AT force fields parametrizations.

### 2.2. Parametrization Using Molecular Dynamics

Using the same cell geometry (Figure 1) and atomic positions as in the QM SCF calculations, we built the Molecular Dynamics (MD) simulations for each cell tested in QM. We assigned multiple combinations of parameters ($\epsilon_{s}$ and $Z_{s}$) using an n-dimensional grid set of parameters, where n is the number of parameters to be determined, and where points in each dimension are evenly-spaced since we wanted to guarantee a good spread of parameters over the testing intervals, as opposed to the Monte Carlo sampling which might not produce the desired spreading effect, when more parameters need to be determined and fewer points can be tested in the testing intervals due to computing capabilities limitations.

After running MD simulations for all combinations of parameters for all lattices simulated in QM, differences between the energy of the ground state and the energy of other states were calculated. Comparison between the QM energy differences and the MD energy differences was done so parameters set that resulted in the minimum coefficient of determination $r^{2}$ concerning the QM differences were chosen. The parametrization was considered complete if the $r^{2}$ value was high enough, compared to a user predefined threshold (close to 1). In case the set with maximum $r^{2}$ was still below the $r^{2}$ tolerance (0.97 in this work), the above procedure was repeated with a smaller test space centered at the set values related to the maximum $r^{2}$, until the coefficient of determination surpassed the threshold or converged to a maximum value.

### 2.3. Parameters Set Validation through Molecular Dynamics Properties Determination

After the parametrization was finished, we tested the parameters set by determining mechanical and thermal properties via MD simulations and comparing results with the values generated using other potential files/force fields. For other force fields, we performed lattice structure optimization by obtaining the lattice parameters corresponding with the system’s minimum energy at 0 K and 0 bar. Then, we used these lattice parameters to run all comparison simulations (mechanical and thermal). We also used the ELATE software [23] (an open-source online application for analysis and visualization of elastic tensors) to compare the stiffness matrix results for aluminum.

MD simulations for mechanical properties determination consisted of using the ELASTIC LAMMPS input file (input file available in the Examples folder in the LAMMPS package), which performs a series of displacement-controlled loading simulations and outputs the simulated material’s elastic constants. We employed periodic boundary conditions on a 5 × 5 × 5-unit cells volume. Results from running this input file for a temperature of 0 K output the components of the material’s stiffness matrix, bulk modulus, Poison ratio, and shear modulus. The elastic modulus is obtained when running an elongation simulation. In this simulation, we used shrink-wrapped boundary conditions, fixed the atoms at one surface of the simulation volume, and moved the atoms at the opposite end, applying strain along the axis perpendicular to these faces. We executed 100 steps moving atoms at a velocity of 1 Å/ps. Then, we stopped these atoms and waited for 100,000 steps, with a time step of 1 fs through the whole simulation, for the system to relax in the new strained state, at which point we measured the stress along the strained axis. Having a stress/strain relationship, we determined the material’s Young Modulus when this relationship along the strained axis (∂σ/∂ε) was at its maximum value.

Thermal properties were also determined via MD simulations for a large temperature interval that allowed the materials to change phase so that the temperatures at which there were deviations between our force field models’ results and results obtained with potential files from the literature could be shown. It was noticed that, at these temperatures, the contributions to the system’s total energy, coming from other phases and defects, are significant.

For the thermal MD simulations, we used periodic boundary conditions, a Quantum Bath (QTB) associated with an NPH (constant number of atoms, volume, and enthalpy) ensemble and, starting at 0.1 K, we varied the temperature, in 10 K intervals, and waited at each new temperature for 50,000 steps, with a timestep of 0.1 fs. At the last 10,000 timesteps, we took an average of the total energy and potential energy of the system. When the potential energy plot through temperature presents a gap, the corresponding temperature ($T_{\mathrm{LS}}$—limit of superheating) is used to determine the material’s melting point ($T_{M}$) as [24]:

(8) $$\frac{T_{\mathrm{LS}}}{T_{M}}-1=\frac{\ln2}{3}\;$$

The specific heat at constant pressure $C_{p}$ is determined by taking the derivative of the total energy ($E_{\mathrm{TOT}}$) with respect to the system’s temperature (T) and dividing the resulting values by the system’s mass (m) as shown by Equation (9).

(9) $$C_{p}=\frac{1}{m}\frac{{\mathrm{dE}}_{\mathrm{TOT}}}{\mathrm{dT}}$$

The system’s density is determined simply by dividing the system’s mass by its volume at each temperature state. The latent heat of fusion is determined by dividing the value of the total energy gap, present in the system’s heating history, by the system’s mass. Lastly, the system’s expansion coefficient is determined by taking the average of the expansion in the 3 orthogonal axes and dividing that value by the initial box length multiplied by the difference between the current temperature and initial temperature (0.1 K).

Another factor to consider while checking the parametrized force field’s adequacy is the diffusion coefficient. We used a 10 × 10 × 10-unit cells volume with periodic boundary conditions and an NVE (constant number of atoms, volume, and total energy) ensemble. We then varied the temperature and for each temperature, we performed 1,000,000 steps with a time step of 1 fs. The diffusion coefficient (D) for the simulated materials was then determined using Equation (10), with the ensemble average of the mean square displacements over all possible system configurations. This ensemble average was calculated using the states at the last 100,000 simulation timesteps for each simulated temperature. We executed this large number of steps to obtain 〈MSD〉 to ensure that the ergodic hypothesis was applicable.

(10) $$\langle \mathrm{MSD}\rangle =\langle \frac{\sum_{i}{\mathrm{dx}}_{i}^{2}{+\mathrm{dy}}_{i}^{2}{+\mathrm{dz}}_{i}^{2}}{N}\rangle =6\mathrm{Dt}\;$$

where ${\mathrm{dx}}_{i}$, $dy_{i}$, and ${\mathrm{dz}}_{i}$ are the displacements of each atom in the simulation volume at the x, y, and z axis, N is the number of atoms in the simulation volume, and t is the simulation time.

For all the simulations with temperature as the independent variable, we employed a temperature range that was enough to notice phase transitions. For simulations with strain as the independent variable, we employed displacements enough to notice a steady stress-strain evolution and determine the material’s elastic modulus.

We have also performed an MD stability analysis by performing equilibrium simulations for different ensemble types (Figure S1 for aluminum, Figure S2 for nichrome, and Figure S3 for $Ti_{2}C$), at 300.0 K, and an atom swapping analysis (Figure S4). We noticed that the systems remained in an equilibrium state with the newly determined force fields and observed no extraneous atomic clustering that would cause instabilities.

## 3. Results

### 3.1. Ground State Determination

We ran an optimization code with cell relaxation in BURAI and obtained the ground states for the aluminum’s atomic system, having 4.04584 Å as lattice parameter in an FCC cell, for the nichrome’s atomic system, having 3.569 Å as lattice parameter in an FCC cell, and for the MXene’s atomic system, having $a_{0}$ = 3.03417 Å and $c_{0}$ = 2.30990 Å (with a cell height of 10 Å to avoid interference from the top and bottom neighbors flakes in the energy’s calculation) as lattice parameters in a Hexagonal and Trigonal P cell.

### 3.2. LJ + AT Parameters Determination

For the aluminum simulations, we performed a first optimization run using an energy range from 0.001 to 1 eV and 0.001 eV intervals for ϵ, and from 1 to 1000 eV with 1 eV intervals for Z. σ was already determined from the quantum mechanics optimization calculations with cell relaxation. After the 1st run, we obtained a coefficient of determination of $r^{2}=0.9997$, which we considered acceptable.

For the nichrome simulations, we performed a first optimization run using an energy range from 0.1 to 1 eV and 0.1 eV intervals for ϵ, and from 100 eV to 1000 eV with 100 eV intervals for Z. After the 1st run, we obtained a coefficient of determination of $r^{2}=0.92$, which we still wanted to improve further. For the 2nd parameters’ set run, we shortened the energy intervals for $\epsilon_{11}$ from 0.22 eV to 0.40 eV in intervals of 0.02 eV, for $\epsilon_{12}$ from 0.02 eV to 0.20 eV in intervals of 0.02 eV, for $\epsilon_{22}$ from 0.52 eV to 0.70 eV in intervals of 0.02 eV, for $Z_{112}$ from 420 eV to 600 eV in intervals of 20 eV, for $Z_{122}$ from 20 eV to 200 eV in intervals of 20 eV, and for $Z_{222}$ from 120 eV to 300 eV in intervals of 20 eV, where the 1 subscript refers to the Ni atom and the 2 subscript refers to the Cr atom. The best parameters set, was the one that resulted in the largest coefficient of determination: $r^{2}=0.9999$.

For the $Ti_{2}C$ simulations, we performed a first optimization run using an energy range from 0.1 to 0.6 eV and 0.1 eV intervals for ϵ, and from 10 to 50 eV with 10 eV intervals for Z. We differentiated between the top and bottom Ti atoms within the simulation volume so the interaction between the top and bottom atoms would not have the same parameters as for Ti atoms in the same plane, due to differences in electrons density fields. After the 1st run, we obtained a coefficient of determination of $r^{2}=0.72$, which we still wanted to improve further. From the 1st parametrization run, we noticed that the system’s energy was more sensible to $\epsilon_{11}$, $\epsilon_{13}$, and $\epsilon_{33}$ variations. Therefore, we decided to apply smaller increments for the determination of these parameters. However, the strongest pair interaction was the one between the Ti and C atoms ($\epsilon_{12}$ and $\epsilon_{23}$). For the 2^nd^ parameters’ set run, we shortened the energy intervals to 0.13 eV < $\epsilon_{11}=\epsilon_{33}$ < 0.18 eV; 0.6 eV < $\epsilon_{12}=\epsilon_{23}$ < 1.0 eV; 0.1 eV < $\epsilon_{22}$ < 0.6 eV; 0.01 eV < $\epsilon_{13}$ < 0.06 eV; and 10 eV < Z < 50 eV. The best parameters set, was the one that resulted in the largest coefficient of determination: $r^{2}=0.97$. Parametrization results for the materials in this study are reported in Table 1.

We compared simulation results through which we could determine mechanical and thermal properties. We performed these simulations using the newly defined LJ + AT parameters and compared its results to results obtained from the same simulations ran with force fields changed to EAM [25,26,27,28], considered an appropriate force field for aluminum, and to EAM, MEAM, and ADP [29,30], considered appropriate force fields for nichrome.

The first comparison, in Figure 2 shows that the energy gaps calculated in MD using the EAM, MEAM, and ADP force fields deviate from energy gaps calculated via QM. The energy determination for the larger strains (>2%) in QM is not considering the occurrence of defects and phase changes since the QM modeling is executed considering a single atomic structure and determines systems energies by altering the lattice parameters. If the atomic configuration changes due to the presence of defects or phase changes, the LJ + AT developed force field model cannot predict the system’s energy accurately anymore, restricting this parametrization scheme’s transferability. The force fields obtained from the cited literature account for defects and phase changes since different configurations from the configuration space are employed in the parametrization process for those models, causing the discrepancies in energy gaps at large strains. Therefore, simulations’ representativity, when employing the newly parametrized LJ + AT force fields, is not expected to be strong for states where the system is excessively strained or temperatures are excessively high.

### 3.3. Mechanical and Thermal Properties Determination

Elastic constants’ determination for the Nichrome alloy was not consistent through the different employed force fields. The reason for that might be that the atomic system output MD data’s signal to noise ratio, is high for this atomic system, as opposed to the Aluminum’s results in Table 2.

The MD results for tensile testing indicate that the LJ + AT, EAM FS, and EAM alloy 99 parameters represent well the elastic portion of the stress-strain curve for the pure aluminum since the curves’ slope approach to aluminum’s experimental expected Young modulus.

Table 2 indicates that the EAM 99 force field model developed by Mishin et al. [26] presents deviations from experimentally determined thermal and temperature-dependent properties because the parameters set fitting for this model was completed with ab initio calculations results, empirically determined equilibrium lattice parameter, cohesive energy, elastic constants $c_{11}$, $c_{12}$, and $c_{44}$, and the vacancy formation energy. They noticed that their ab initio calculations underestimated the lattice parameter of aluminum at an equilibrium configuration. To fix this deviation from the lattice parameters’ experimental value, they applied a multiplicative correction factor to the interatomic distances before calculating the system’s energy. Additionally, their parametrization scheme used different possible atomic configurations in the aluminum’s configuration space, improving its transferability. When the focus is on minimizing errors for specific properties prediction, the resulting model parameters may not minimize errors for other disregarded or less-weighed properties during the parameters’ fitting. This explains the large deviations from experimental values for thermal- and temperature-dependent properties determined via the EAM 99 potential. EAM JNP, as well as the herein-developed LJ + AT models, presented similar deviations from the experimental values for all types of properties, although also presenting some good matching results for some properties, because those models only rely on ab initio calculations to derive force field model parameters, and ab initio calculation results may deviate from experimental results, as verified by Mishin et al. [26]. However, those errors may be considered non-critical for an initial material assessment. Mendelev et al. [27,28] developed a Finnis–Sinclair EAM force field model using the perfect crystal lattice parameter, cohesive energy, elastic constants, point defect formation energies, and solid–liquid equilibrium properties experimentally determined to optimize their parametrization scheme, making predictions accurate for mechanical properties and properties at phase transitions. This made simulations employing this force field present the best match with experimental results throughout all the material properties considered in this work. Although semi-empirically parametrized force field models present a good match with experimental results, they use experimental results to guide their parameters’ fitting. The principal aspect of this work is producing a force field model capable of satisfactorily characterizing materials employing simple models without the aid of experimental results. Therefore, those semi-empirical force field models were used to show how close the LJ + AT simulations results were to their results. The EAM JNP potential was used to show how a fully simulations-based force field model may provide results that deviate from experimentally determined properties and how our model results errors are compared to the EAM JNP errors.

**Figure 3:**
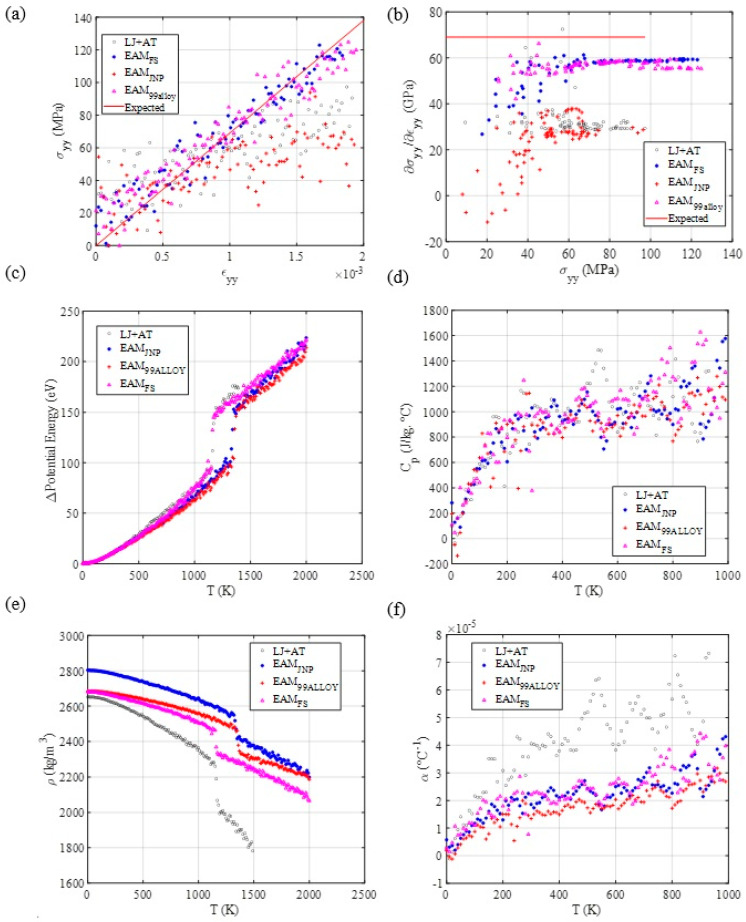
MD mechanical response for tensile testing and thermal response using different aluminum force fields. (**a**) Tensile testing. (**b**) Stress-strain curve’s slope as a function of stress, as an indicator of yield point. (**c**) Potential energy, (**d**) Specific heat, (**e**) mass density, and (**f**) thermal expansion coefficient evolution through temperature.

The thermal MD simulation results (Figure 3), indicate that all employed force fields show similar specific heat at constant pressure and similar potential energy evolution through a large temperature interval, where most of the material maintained its starting phase. However, thermal expansion-related properties (thermal expansion coefficient—α and mass density—ρ) can have significantly deviating results. This is mostly explained by the different starting lattice parameters (at T≈0 K). A larger deviation at larger temperatures for the LJ + AT, when compared with the other EAM potentials, is expected because of the lack of other phases and defects in the parametrization process. Although these differences are present at larger temperatures, the LJ + AT force fields still yielded acceptable results for aluminum’s properties close to room temperature (RT).

The MD results for tensile testing indicate that the LJ + AT, MEAM, and APD parameters represent well the elastic portion of the stress–strain curve for the nichrome alloy since the curves’ slope approach nichrome’s experimentally expected Young modulus. However, other elastic constants may be more accurately determined via MEAM potentials.

**Figure 4:**
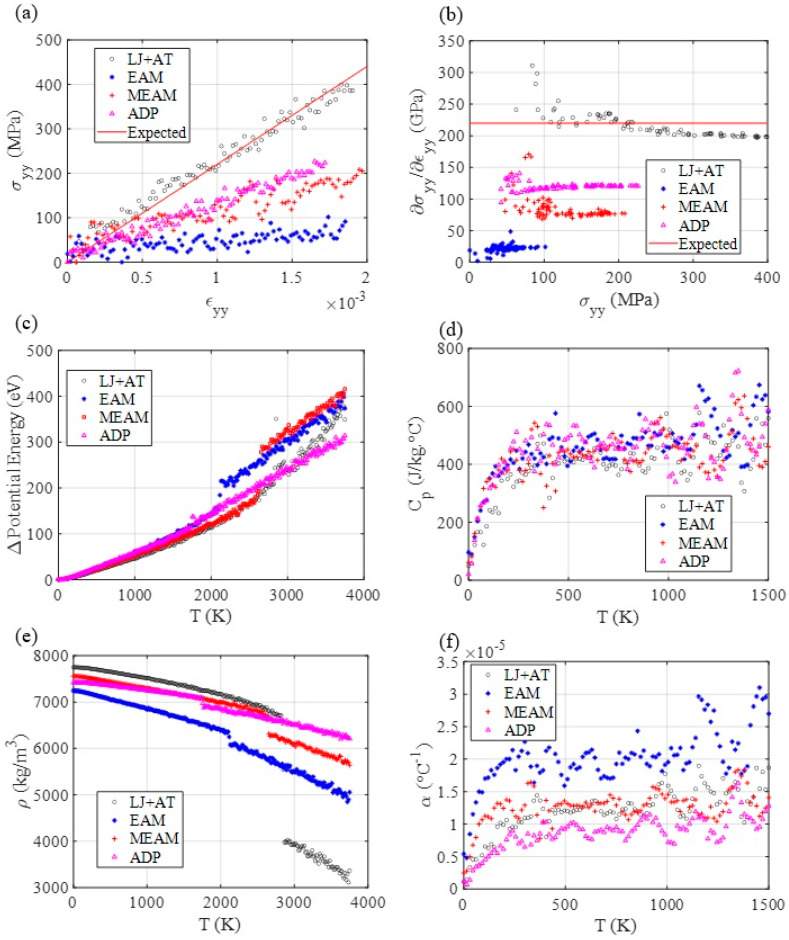
MD mechanical response for tensile testing and thermal response using different nichrome force fields. (**a**) Tensile testing. (**b**) Stress-strain curve’s slope as a function of stress, as an indicator of yield point. (**c**) Potential energy, (**d**) Specific heat, (**e**) mass density, and (**f**) thermal expansion coefficient evolution through temperature.

The thermal MD simulation results (Figure 4), indicate that all employed force fields show similar specific heat at constant pressure and similar potential energy evolution through a large temperature interval, where most of the material maintained its starting phase. Differently from aluminum, nichrome’s parametrization via LJ + AT showed results comparable to the most used force fields, and to experimental values at the solid phase [31]. A reason for this is the more abrupt phase transition, closer to the melting pointy for this alloy. Therefore, the starting phase persists for longer, making the predictions via the LJ + AT parameters more accurate since the resulting thermal simulation values are close to the values obtained with the MEAM force field and within the range of experimental results (Table 3).

We expect the $Ti_{2}C$ MXene’s thermal and mechanical properties, determined via MD simulations, using the LJ + AT parameters, to be accurate up to the temperatures and strains where the material’s stability can’t be further guaranteed, as were the pure aluminum’s, and the nichrome’s cases.

**Figure 5:**
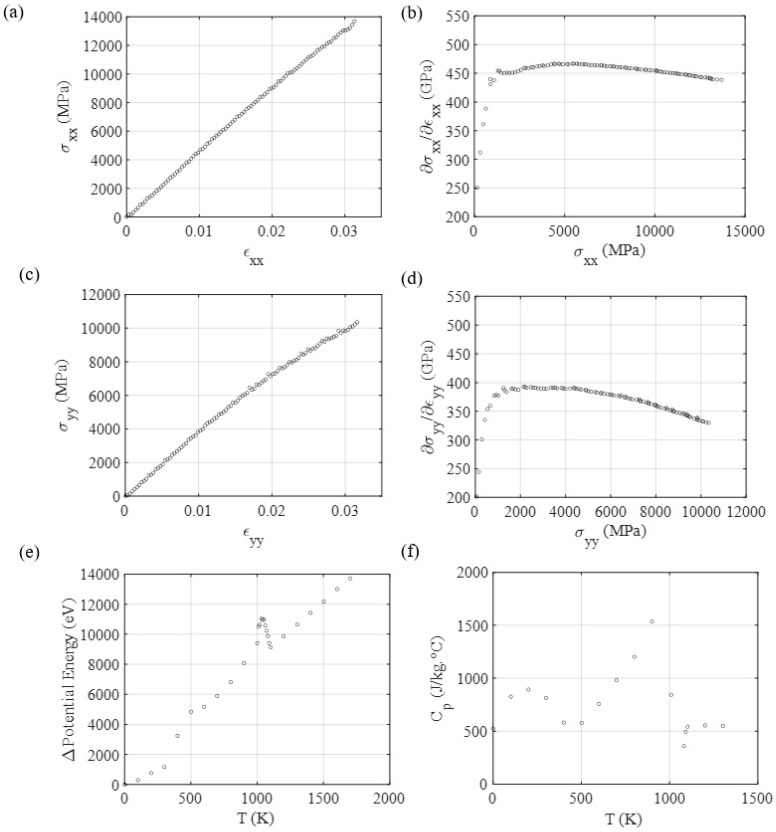
MD mechanical response for tensile testing over ${\mathrm{Ti}}_{2}C$ MXene, using the parameters determined via the LJ + AT parametrization process, applied to different orientations (zigzag X and armchair Y directions). (**a**) Tensile testing for loads applied at the zigzag direction. (**b**) Stress-strain curve’s slope as a function of stress, as an indicator of yield point for the zigzag direction. (**c**) Tensile testing for loads applied at the armchair direction. (**d**) Stress-strain curve’s slope as a function of stress, as an indicator of yield point for the armchair direction. (**e**) Potential energy, and (**f**) Specific heat evolution through temperature.

The MD results for tensile testing, shown in Figure 5, where the *X*-axis is the zigzag direction, and the *Y*-axis is the armchair direction, show that the Young modulus for the $Ti_{2}C$ MXene at the zigzag direction is 465 GPa and for the armchair direction, it is 397 GPa. The Poisson ratio was determined as ν≈0.30 for both directions. LJ + AT parameters give a good estimation for the Young modulus of $Ti_{2}C$ MXene, when we compare this work’s results with results from QM simulations performed by Kurtoglu et al. [32]. In their work, they obtained $c_{11}$ = 636 GPa. If the material is considered isotropic, the Young modulus can be determined as: $E_{Ti_{2}C}=c_{11}\left(1+\nu \right)\left(1-2\nu \right)/\left(1-\nu \right)\approx 467$ GPa), which has a small error when compared with the value for the zigzag direction. If we assume that $Ti_{3}C_{2}$ is also isotropic and has the same poison ratio as $Ti_{2}C$, using $c_{11}=523$ GPa [32], we would obtain $E_{Ti_{3}C_{2}}=384$ GPa, which is close to the empirically determined value of $E_{Ti_{3}C_{2}T_{x}}=330$ GPa [33]. The deviation between the simulated and experimentally obtained Young modulus values for $Ti_{3}C_{2}T_{x}$ can be explained by the presence of point defect occurrences in the real flakes, while the simulated ones do not account for defects.

**Figure 6:**
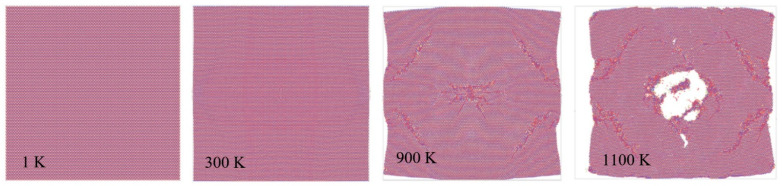
${\mathrm{Ti}}_{2}C$ MXene’s thermal stability MD simulation employing the hybrid LJ + AT force field parameters. Images were obtained using OVITO [36] (atomic systems visualization software).

MD simulations for thermal stability (Figure 6) indicate that ${\mathrm{Ti}}_{2}C$ is thermally stable, without significant occurrences of structural changes and defects until 1000 K, which is in good agreement with the simulation results obtained by Borysiuk and Moshalin [34] and experimental results obtained by Wyatt et al. [35], where they found that between 900 °C and 1200 °C, a new phase ($TiC_{y}$) is formed.

### 3.4. Diffusion Coefficient

**Figure 7:**
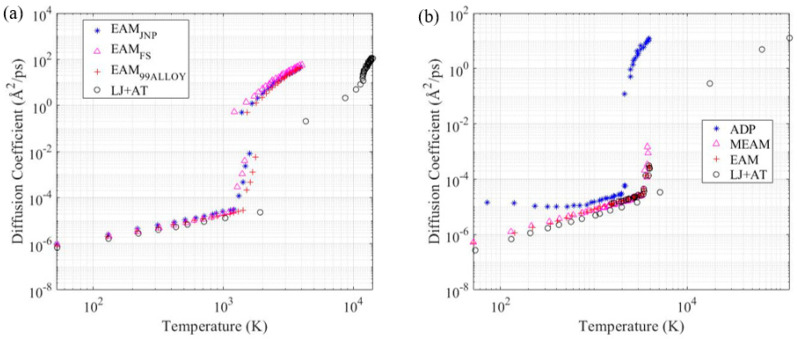
Diffusion coefficient as a function of temperature in a log-log scaled plot for (**a**) aluminum, and (**b**) nichrome.

As we expected, from what was seen in the temperature-dependent thermal properties, the diffusion coefficient determined using the Lennard-Jones combined with the Axilrod-Teller parameters set diverged from the coefficient determined with other established force fields after considerable phase change had happened. However, the values for the diffusion coefficient were approximate for all the force fields at the temperatures where no significant phase transformation happened. Such results match, especially comparing the LJ + AT force field model results with the EAM FS results, confirm the LJ + AT validity for the aluminum’s diffusion coefficient determination. Mendelev et al. [27] verified that the EAM FS parametrization presents a good match with the aluminum’s experimental diffusivity. This helps to prove the fact that the force fields parameters set determined with the 2 and 3-body energy terms, proposed in this work, should be restricted to strain states and temperature ranges where no significant phase changes take place. The developed simplified force field model can satisfactorily simulate the system when the predominant phase is the one used for the model’s parametrization. Therefore, the model predictions may present an increased error for temperatures and strain states outside of the parametrization range, as can be seen in Figure 7, at temperatures above the melting point, where the LJ + AT model predictions highly deviate from other known and effective literature force field models.

## 4. Discussion

After two parametrization optimization runs, we noticed that the systems’ energy variations, when varying the simulation volumes and atomic positions, determined via MD simulations present an acceptable fitting considering the system’s energy determined via QM simulations. The coefficient of determination for the MD model compared with the QM model was $r^{2}\geq 0.97$ for the aluminum’s, nichrome’s, and MXene’s parametrizations.

Simulated material properties results, show that most mechanical and thermal properties obtained using LJ + AT force fields are fair approximations to values obtained with other force fields, and in fair proximity with experiment results (Table 2 and Table 3). Then the LJ + AT parametrization approach shows its relevance as a tool for an initial novel material’s mechanical and thermal behavior assessment via MD simulations. This fact could also be confirmed for the $Ti_{2}C$ for which the elastic modulus determined via MD with the LJ + AT parameters was −0.4% off the QM determined value [32].

Although the simulation results show that the LJ + AT force field can generate fair material properties predictions for pure metals, alloys, and 2D materials, by producing results that are similar to those obtained with popularly used force fields, its application should be restricted to conditions where excessive phase changes and defects are not present. If good accuracy is expected for phase changes and defects, the parametrization process should be repeated for those other phases and different phase interactions, making the parametrization process more computationally expensive. Although a simple guide for initial MD simulations to predict novel materials properties is desired, the accuracy of results shall only be considered satisfactory for a range of temperatures and strains where the material’s phase remains stable.

The parametrization process depends on whether the number of different types of atoms and interactions present in the system is reduced since these numbers define the dimension of the parametrization array, making the parametrization time exponentially grow with an increasing number of atoms and interaction types in the system. Therefore, parametrization of high entropy alloys may get even more computationally expensive than deriving EAM and MEAM force fields for these materials.

As novel classes of materials (two-dimensional MXenes and ferroelectric metals [37] are being developed and those classes have a reduced number of atom types and interactions, LJ + AT can be an interesting alternative to the highly complex parametrization processes to create ReaxFF, MEAM, or EAM force fields. When novel materials are developed, it is important to promptly determine their properties so they can be better considered as alternatives for other materials currently available. For the specific case of MXenes, the determination of single flakes’ properties (mechanical and thermal) is not easy, making MD and QM simulations important tools for preliminary properties predictions and materials behaviors analysis under specific mechanical and thermal conditions.

For a temperature range that extends above the melting point of the simulated materials (Figure 7), the diffusion coefficient determined with the LJ + AT parameters approached the results for other EAM force fields. However, the parametrization discussed in this work applies, with a satisfactory fidelity level, to the proposed atomic structure only. For higher temperatures, above the phase change point, there may be a level of atomic structure uncertainties that can make the EAM force field a better choice for MD simulations since EAM’s performance is not conditioned to the atomic structure.

## 5. Conclusions

The procedure proposed in this work may considerably shorten force field development for the initial assessment of novel materials’ properties (for example the novel 2D MXenes), for which, a fully open-source force field is not readily available.

It was shown that results obtained with the parametrized Lennard-Jones combined with Axilrod -Teller (LJ + AT) force fields were comparable to results obtained with parameters files available in the literature and experimental results if no significant phase changes are present. Such proximity can make this proposed parametrization methodology an option for researchers who need to conduct simulation-based research of novel bulk metallic and 2D materials but cannot find appropriate force field parameters in the literature and do not have expensive computational resources available. Adding to this, the parametrization model is mathematically simple and can be computationally inexpensive if a reduced number of 2 and 3-body interactions is present.

## Figures and Tables

**Table 1:** Parameters for Aluminum’s, Nichrome’s, and $Ti_{2}C$ MXene’s hybrid LJ + AT force fields.

Material	Parameter	Value	Unit
Aluminum	ϵ	0.168	eV
	σ	2.5487	Å
	Z	236	eV
Nichrome	$\epsilon_{11}$	0.36	eV
	$\sigma_{11}$	3.1796	Å
	$\epsilon_{12}$	0.12	eV
	$\sigma_{12}$	2.2483	Å
	$\epsilon_{22}$	0.58	eV
	$\sigma_{22}$	2.2483	Å
	$Z_{112}$	540	eV
	$Z_{122}$	80	eV
	$Z_{222}$	240	eV
$Ti_{2}C$	$\epsilon_{11}$	0.18	eV
	$\sigma_{11}$	2.7031	Å
	$\epsilon_{12}$	0.6	eV
	$\sigma_{12}$	1.8693	Å
	$\epsilon_{13}$	0.01	eV
	$\sigma_{13}$	2.5827	Å
	$\epsilon_{22}$	0.5	eV
	$\sigma_{22}$	2.7031	Å
	$\epsilon_{23}$	0.6	eV
	$\sigma_{23}$	1.8693	Å
	$\epsilon_{33}$	0.18	eV
	$\sigma_{33}$	2.7031	Å
	$Z_{112}$	10	eV
	$Z_{233}$	10	eV
	$Z_{122}$	10	eV
	$Z_{223}$	10	eV
	$Z_{111}$	10	eV
	$Z_{333}$	10	eV

**Table 2:** Mechanical and thermal properties of aluminum determined via MD using different force fields at 300 K.

Property	LJ + AT	EAM 99	EAM FS	EAM JNP	ELATE	Experimental [26]
C11 (GPa)	107.2695	113.7967	105.0917	111.3806	104	114
C12 (GPa)	70.8877	61.5546	59.4629	85.1381	73	61.9
C44 (GPa)	56.3227	31.5946	30.6588	45.9262	32	31.6
Bulk Modulus (GPa)	83.0150	78.9683	74.6725	93.8857	83	79
Young (GPa)	72.4258	66.1612	61.2460	38.0122	65.4847	70
Poisson Ratio	0.3979	0.3510	0.3614	0.4332	0.37	0.35
Shear Modulus (GPa)	37.2568	28.8576	26.7366	29.5237	23.6667	26
Latent Heat of Fusion (kJ/kg)	405.86	345.43	360.81	371.18	---	396
Melting point (K)	934.16	1096.6	934.16	1072.3	---	933
$\mathrm{Density}\;\mathrm{at}\;\mathrm{RT}\;(\mathrm{kg}/m^{3}$)	2601	2663	2645	2774	---	2700
$C_{p}$ at RT (J/kg·℃ )	1049	804.3	962.6	841.3	---	921
$\alpha \;\mathrm{at}\;\mathrm{RT}\;(10^{-6}$$)\;({℃}^{-1}$)	40.6	13.4	22.1	18.6	---	23

**Table 3:** Mechanical and thermal properties of nichrome determined via MD using different force fields at 300 K.

Property	LJ + AT	EAM	MEAM	ADP	Experimental [31]
C11 (GPa)	437.3272	111.15091	315.5455	143.4087	---
C12 (GPa)	216.3067	98.4087	129.8099	144.2840	---
C44 (GPa)	161.6199	83.1493	99.1138	91.4083	---
Bulk Modulus (GPa)	289.9802	102.6561	191.7217	143.9923	110–205
Young (GPa)	311.0619	48.7332	170.8231	140.5154	150–245
Poisson Ratio	0.3309	0.4696	0.2915	0.4990	0.26–0.325
Shear Modulus (GPa)	136.0651	44.7602	95.9908	45.4853	55–100
Latent Heat of Fusion (kJ/kg)	328.62	225.79	303.46	90.96	275–320
Melting point (K)	2290.7	1705.9	2144.5	1413.4	1475–1710
$\mathrm{Density}\;\mathrm{at}\;\mathrm{RT}\;(\mathrm{kg}/m^{3}$)	7704	7147	7493	7385	7750–8650
$C_{p}$ at RT (J/kg·℃)	376.9	467.7	401.9	419.2	380–500
$\alpha \;\mathrm{at}\;\mathrm{RT}\;(10^{-6}$$)\;({℃}^{-1}$)	10.94	22.69	12.91	6.47	9–16

## Data Availability

The data supporting the findings of this study are available within the article.

## Supplementary Materials

The following are available online at https://www.mdpi.com/article/10.3390/ma14216352/s1, Figure S1: Total energy through the aluminum’s MD equilibrium simulations, using the derived LJ+AT potentials for aluminum, at the initial temperature of 300.0 K. (a) NPT ensemble, (b) NVT ensemble, and (c) NVE ensemble, Figure S2: Total energy through the nichrome’s MD equilibrium simulations, using the derived LJ+AT potentials for aluminum, at the initial temperature of 300.0 K. (a) NPT ensemble, (b) NVT ensemble, and (c) NVE ensemble, Figure S3: Total energy through the Ti_2_C MD equilibrium simulations, using the derived LJ+AT potentials for aluminum, at the initial temperature of 300.0 K. (a) NPT ensemble, (b) NVT ensemble, and (c) NVE ensemble, Figure S4: The radial distribution function for the simulations using the Lennard-Jones and Axilrod-Teller parameters overlayed with results obtained after employing the MEAM force field. Both simulations considered atoms swapping.
