# Supplementary material for: Employing Hybrid Lennard-Jones and Axilrod-Teller Potentials to Parametrize Force Fields for the Simulation of Materials’ Properties
Source: Materials (Basel). 2021 Oct 24;14(21):6352. doi: 10.3390/ma14216352 (PMC8585255; doi:10.3390/ma14216352)
Supplement: Supplementary file 1 [file materials-14-06352-s001.zip › materials-1356718-supplementary.pdf]

materials

MDPI

Supplementary Material

Employing Hybrid Lennard-Jones and Axilrod–Teller Potentials to Parametrize Force Fields for the Simulation of Materials' Properties

Danilo de Camargo Branco [1] and Gary J. Cheng [2,*]

[1] School of Aeronautics and Astronautics, Purdue University, West Lafayette, IN 47907, USA
[2] School of Industrial Engineering, Purdue University, West Lafayette, IN 47907, USA
* Correspondence: gjcheng@purdue.edu

Citation: Branco, D.d.C.; Cheng, G.J. Employing Hybrid Lennard-Jones and Axilrod–Teller Potentials to Parametrize Force Fields for the Simulation of Materials' Properties. *Materials* **2021**, *14*, 6352. https://doi.org/10.3390/ma14216352

Academic Editors: Paolo Restuccia and James Ren

Received: 10 August 2021
Accepted: 21 October 2021
Published: 24 October 2021

Publisher's Note: MDPI stays neutral with regard to jurisdictional claims in published maps and institutional affiliations.

Copyright: © 2021 by the authors. Licensee MDPI, Basel, Switzerland. This article is an open access article distributed under the terms and conditions of the Creative Commons Attribution (CC BY) license (https://creativecommons.org/licenses/by/4.0/).

S1. Energy evolution in an equilibrium state

One way to check if the LJ+AT parameters are appropriate for its material is to test it in equilibrium Molecular Dynamics (MD) simulations using different ensembles. For each material studied (Aluminum, Nichrome and Ti_2C) we have performed a simulation to check if the total system energy was kept constant through the simulation timesteps.

We used periodic boundary conditions and a simulation volume containing 10 x 10 x 10 aluminum unit cells. We then applied the LJ+AT parameters from Table 1 in the main text. We used a time step of 1fs and an initial temperature of 300.0 K. In the first simulation case, we used an NVE ensemble (constant number of atoms, volume, and total energy). In the second simulation case, we used an NVT ensemble (constant number of atoms, volume, and temperature). In the third simulation case, we used an NPT ensemble (constant number of atoms, pressure, and temperature). In this last case, the NPT ensemble considered the "aniso" keyword for the barostat. This keyword makes the simulation consider barostating the x, y, and z-directions independently, allowing the simulation box to change its three dimensions freely.

The total energy graphs through timestep are depicted in Figure S1 for aluminum simulations, Figure S2 for nichrome simulations, and in Figure S3 for $\mathrm{Ti_2C}$. It can be seen that the total energy was kept without significant variations, as expected for the NPT (Figure S1 (a) for aluminum, Figure S2 (a) for nichrome, and Figure S3 (a) for $\mathrm{Ti_2C}$), NVT (Figure S1 (b) for aluminum, Figure S2 (b) for nichrome, and Figure S3 (b) for $\mathrm{Ti_2C}$), and NVE (Figure S1 (c) for aluminum, Figure S2 (c) for nichrome, and Figure S3 (c) for $\mathrm{Ti_2C}$) cases.

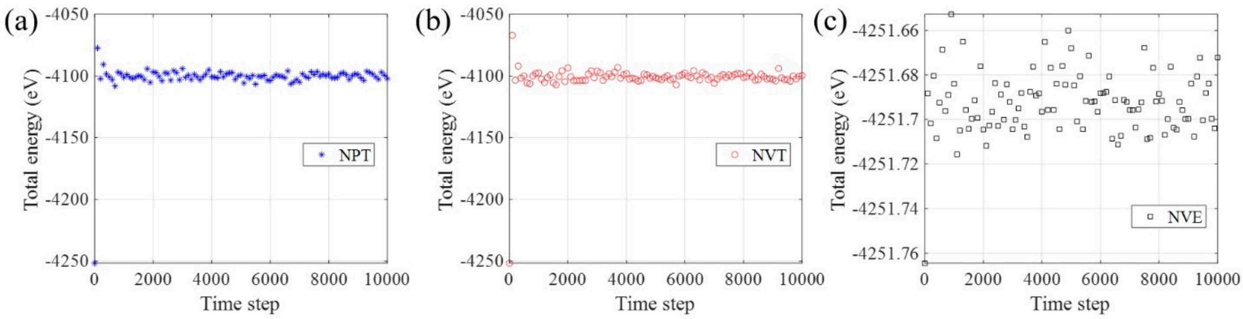

Figure S1. Total energy through the aluminum's MD equilibrium simulations, using the derived LJ+AT potentials for aluminum, at the initial temperature of 300.0 K. (a) NPT ensemble, (b) NVT ensemble, and (c) NVE ensemble.

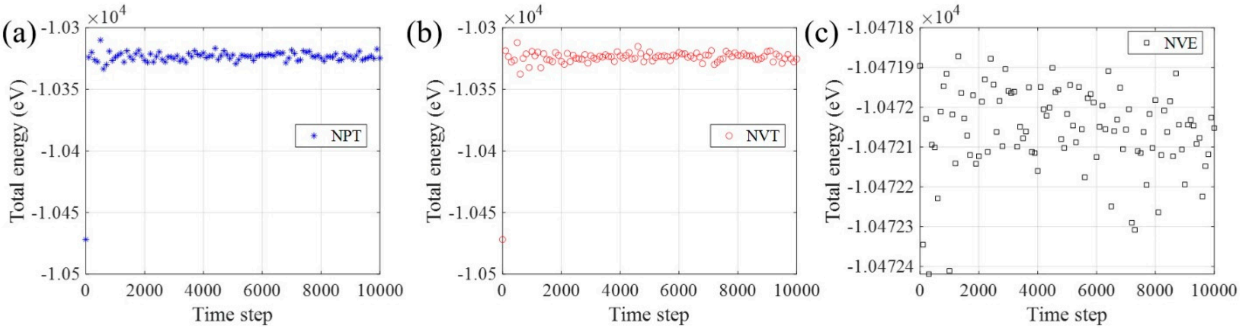

Figure S2. Total energy through the nichrome's MD equilibrium simulations, using the derived LJ+AT potentials for nichrome, at the initial temperature of 300.0 K. (a) NPT ensemble, (b) NVT ensemble, and (c) NVE ensemble.

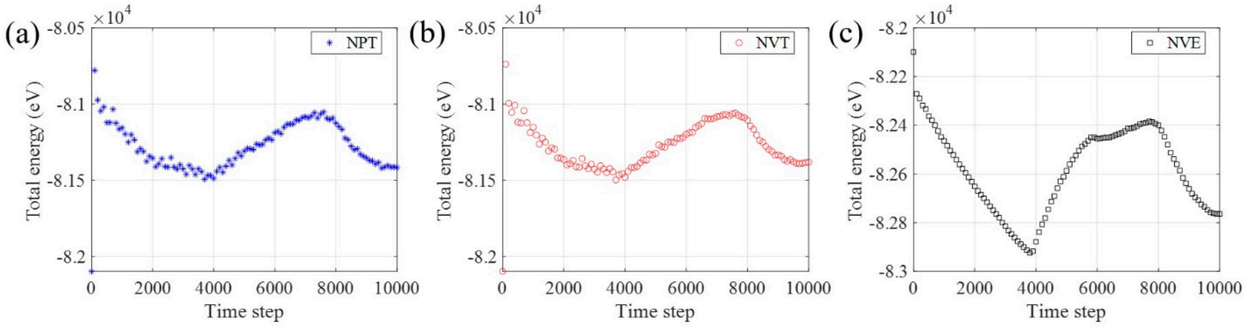

Figure S3. Total energy through the Ti_2C MD equilibrium simulations, using the derived LJ+AT potentials for Ti_2C, at the initial temperature of 300.0 K. (a) NPT ensemble, (b) NVT ensemble, and (c) NVE ensemble.

The differences in the equilibrium energy for the NPT and NVT cases compared with the NVE case is due to the fact those simulations (NPT and NVT) considered the lattice parameter determined in the Quantum Mechanical (QM) simulations, where its determination was done under the 0 K consideration, as one initial geometrical condition. Therefore, NPT and NVT simulation ensembles adjust the simulation volume dimensions and the simulation pressure, respectively, before reaching equilibrium, which causes the total energy at the equilibrium to differ from the initial total energy, while NVE simulations will adjust the simulation temperature, maintaining the total energy constant.

We noticed that the parametrized force fields for the aluminum, nichrome, and Ti_2C allowed the simulated materials to converge to an equilibrium state, proving that the proposed force fields can simulate the materials at an equilibrium state, using different ensembles, and obtain a result that does not diverge from reality.

S2. Effect of atomic structure change via atom/swap LAMMPS feature

After performing the thermal equilibrium simulations, we decided to repeat the NPT ensemble simulation for nichrome (this would not work for aluminum since aluminum contains only one atom type) and add a fix for atoms swap (that uses the Monte Carlo model for swapping atoms with a probability determined via the Metropolis criterion). This simulation was performed to check how different atomic configurations (formed by substitutional defects) would affect the system's equilibrium state.

We applied the atoms swap fix for 1000 steps, at the beginning of the simulation until around 110 atoms were successfully swapped from the 4000 total atoms in the simulation volume. After that, we run 100,000 simulation steps to guarantee that the system reached its equilibrium state.

Figure S4 shows that the radial distribution function with the atoms swapping considerations, employing the MEAM, and the LJ+AT force fields did not significantly diverge from each other at the last simulation step. This indicates that no unexpected clustering of structural instability happens in the system when using the Lennard-Jones and Axilrod-Teller force field parameters. The fact that the LJ+AT simulation presented less sharp peaks, and larger local minima for smaller radii, compared to the MEAM simulations, indicate that the LJ+AT simulations presented a slightly larger disorder than MEAM, but not at a level that would cause system instabilities.

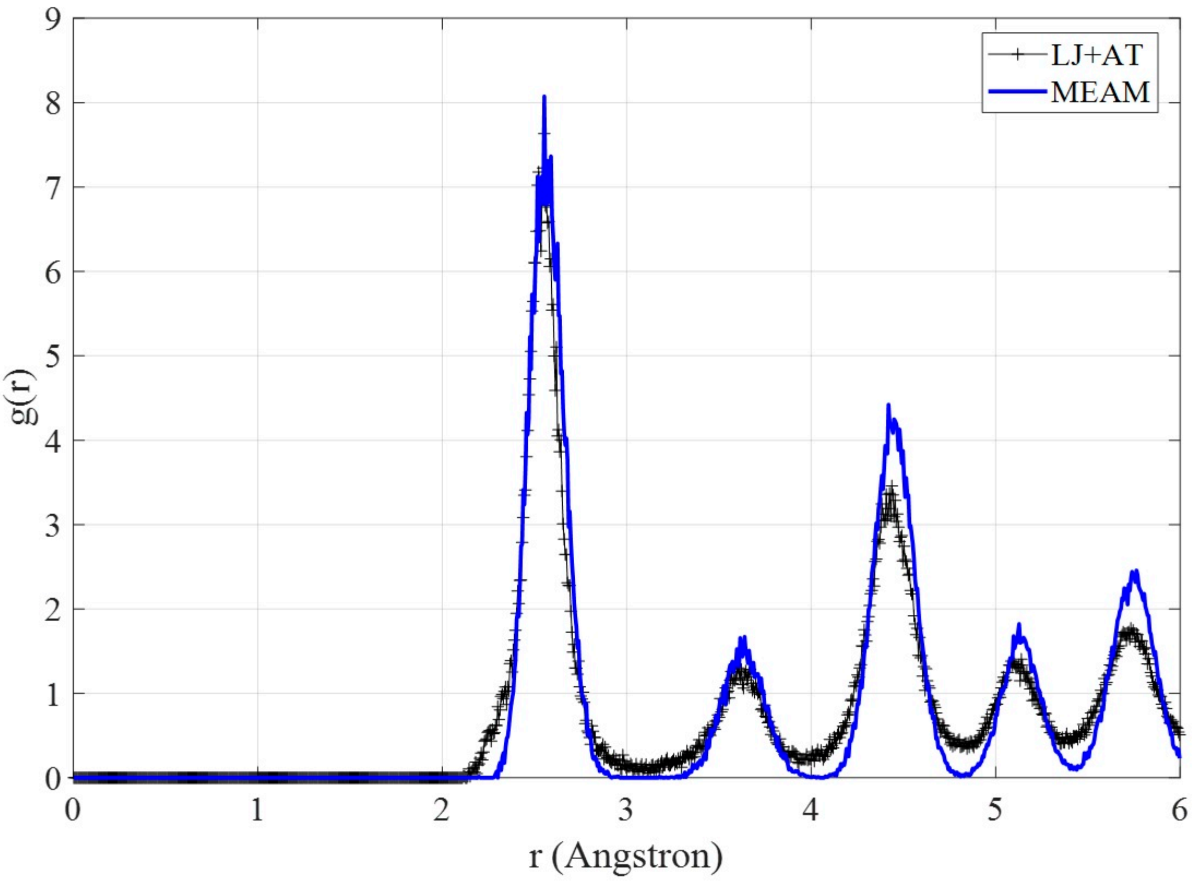

Figure S4. The radial distribution function for the simulations using the Lennard-Jones and Axilrod-Teller parameters overlayed with results obtained after employing the MEAM force field. Both simulations considered atoms swapping.
